# Ray-Based Physical Modeling and Simulation of Multibeam Sonar for Underwater Robotics in ROS-Gazebo Framework

**DOI:** 10.3390/s25051516

**Published:** 2025-02-28

**Authors:** Woen-Sug Choi

**Affiliations:** Department of Ocean Engineering, Korea Maritime and Ocean University, Busan 49112, Republic of Korea; wschoi@kmou.ac.kr

**Keywords:** CUDA acceleration, multibeam sonar, underwater robotics, real-time simulation, ROS-gazebo

## Abstract

While sonar sensors are crucial for underwater robotics perception, the key challenge lies in traditional multibeam sonar simulation’s lack of comprehensive physics-based interaction models. Such missing physical aspects lead to sonar imagery discrepancies, such as the absence of coherent imaging systems and speckle noise effects exposing risks of over-fitted control designs of the systems using the sonar perceptions. Previous research addressed this gap by introducing a physics-based simulation approach by direct calculation of the point-scattering model equations from perception data obtained from rasterization. However, the raster-based method could not control the resolution of data to pipeline into image generation, and its limitation was explicitly presented in local search scenarios where the distance between data is large. To eliminate those limitations and extend capabilities without losing the quality of the image, this paper introduces a ray-based approach to replace the raster-based method when obtaining the perception data from the simulated world to pipeline into physical equation calculations. The results of the ray-based and raster-based models are compared for the front floating object and the ground grazing local search scenario to confirm that the ray-based method maintains equal quality of sonar image generation, including physical characteristics, but it has more flexibility and capability in control of data resolution for correct sonar image generation.

## 1. Introduction

Utilizing virtual environments for simulating robotic systems has emerged as a crucial methodology for assessing operational integrity and performance. The underwater domain presents unique perceptual challenges for robotic systems, including turbidity, backscatter, and low light conditions [1]. In these environments, sonar-based systems frequently arise as the primary, if not sole, reliable means of perception [2]. This reality necessitates developing and refining high-fidelity sonar sensor models to enhance their accuracy and those of the virtual simulations. The fidelity of these models plays a critical role in bridging the gap between simulated environments and real-world conditions, thereby improving the transferability of control strategies and algorithms developed in silico to actual underwater robotic systems.

Creating accurate sonar perception models that simulate high-frequency sound propagation poses significant computational challenges. While true-to-life acoustic data calculations often exceed the computational resources available for real-time robotic simulations, the development and application of synthetic models to emulate this data have emerged as a promising avenue of research. These synthetic models aim to balance computational efficiency and perceptual accuracy, enabling real-time simulations that capture the essential characteristics of sonar-based perception in underwater environments.

While the sonar is essential and one of the most crucial perception tools for underwater robotics, the methods incorporated in the recently released underwater simulators in ROS-Gazebo framework [3,4] only included basic calculations of projecting 3D point clouds virtual scenes into top-view 2D image [2,5,6,7]. The main goal of those approaches was to correctly capture the occluded shadow behind the object from the sonar’s point of view. The ray-based methods are commonly used to detect object or boundaries that comes into contact [8,9] and developed into ray-tracing methods [10,11]. The most recent underwater robotics framework was developed in Unreal Engine [12,13] to incorporate multi-path reflections using ray-tracing and added acoustics calculations and noises to represent the sonar image better. Some researchers have attempted to incorporate generative adversarial networks [14,15], style transfer networks [16], and various methods incorporting neural network [17,18,19] to recreate more authentic noise in the sonar image. Such implementations do not consider any physical aspects of underwater acoustics.

While these simulation approaches represent significant advances in underwater robotics, each exhibits fundamental limitations in modeling the complex physics of sonar-environment interactions. The ROS-Gazebo implementations, while computationally efficient, oversimplify acoustic interactions by reducing them to basic geometric projections. Similarly, although the Unreal Engine’s ray-tracing capability adds multi-path reflections, it falls short of modeling crucial acoustic phenomena such as frequency-dependent attenuation, multiple scattering effects, and complex material interactions. Learning-based approaches using GANs and style transfer networks can reproduce realistic-looking noise patterns but lack the physical principles necessary for reliable simulation of acoustic behavior in varying environmental conditions.

The most advanced research to improve sonar image generation while satisfying the requirements of robotics to produce the image with a high refresh rate was attempted by Cerqueira et al. [20,21] by adopting the image processing of the projected 2D image from the 3D point cloud with pre-calculated acoustics parameters. These methods use pre-computed acoustic parameters in an image-processing manner, incorporating the echo intensity, pulse distance, and field-of-view components. While this approach represents a significant advancement in computational efficiency, its pixel-by-pixel manipulation of sonar images introduces fundamental limitations in accurately representing real-world sonar behavior.

Specifically, the simplified image processing approach struggles to reproduce changes due to various sonar specifications and operation frequency modes such as speckle noise patterns and time–angle ambiguities inherent in pulse–echo imaging systems. Given these inherent constraints, more deterministic approaches, although simplified, in the representation of acoustics calculations are needed to address these limitations and provide more realistic simulations of various sonar models.

Previous work by Choi, et al. [22], which was part of the Dave [23] that inherited the UUV Simulator [2] on ROS-Gazebo framework, explored the calculation of beam-level time series sonar data. A point-based scattering model [24] was employed alongside CUDA parallel computing to manage the high computational demand. This approach successfully produced realistic sonar images at practical refresh rates for robotic simulations, including raw sonar data analogous to that from actual sonar hardware.

The employed methodology in the previous work [22] exhibited inherent limitations, primarily due to its reliance on 2D rasterized scenes derived from the 3D virtual environment. This approach utilized the generic depth camera plugin of Gazebo to acquire depth image data. Rasterization, a technique commonly used in computer graphics, converts 3D objects into a 2D-pixel grid. In this context, each pixel in the rasterized scene was treated as a point scatterer. Although perception data from the 2D rasterization does not resemble the real-world beam emission physics, it was simplistic approach to implement the methods into Gazebo simulation. This led to diminished resolution for objects at greater distances from the sonar sensor and non-uniform spacing between point scatterers.

To address these raster-based method constraints, a ray-based data input method was developed. In contrast to rasterization, the ray-based method simulates the path of sound as it interacts with virtual objects. This approach more accurately represents the average direction of transducer beams and their interactions with objects within the main lobe of the directivity pattern.

The ray-based method offers several advantages over rasterization in sonar simulation while maintaining the equal quality of the sonar image generation capability incorporation physical aspects:

- Improved distance accuracy—Ray tracing calculates precise intersection points with 3D objects, avoiding the discretization errors inherent in rasterization.
- Scalable resolution—The number of rays can be adjusted to balance between accuracy and computational cost, unlike the fixed resolution of a rasterized image.
- Enhanced directivity pattern calculations—The ray-based approach allows for more accurate applications of directivity pattern interference calculations, leading to more realistic beam behavior modeling.

Consequently, the ray-based method enhances the simulation fidelity by providing a more precise representation of the acoustic environment and beam-object interactions. This improvement more accurately represents complex underwater scenes through fully three-dimensional calculations from data acquisition to beam-ray interactions, while offering controllable perception resolution. Its benefit is particularly significant for local search scenarios [25,26] where high resolution is required, one of the critical scenarios for developing robust underwater robotic systems.

In this paper, the ray-based method is explained compared with the raster-based method. Thereafter, simple comparisons between real-world and simulated data is performed for quantitative validations of the embedded physical calculation methods. Finally, the results of the ray-based and raster-based models are compared for the front floating object and the ground grazing local search scenario to confirm that the ray-based method maintains equal quality of sonar image generation, including physical characteristics, but it has more fidelity and flexibility.

## 2. Methods

### 2.1. Beam-Based Acoustic Calculation

**Figure 1:**
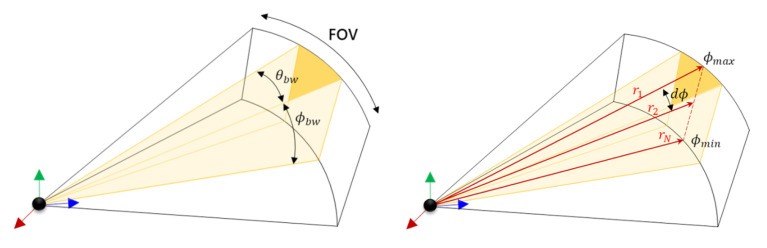
Schematic representation of the calculation unit and Its properties [22] (**Left**: One sonar beam depicted within the Field of View (FOV); **Right**: Multiple rays shown within a single sonar beam).

The fundamental element in the acoustic physics implementation of the model is the ‘beam’ of the physical sonar transducers. The Field of View (FOV) contains a number of beams determined by hardware specifications. As illustrated in Figure 1, each beam within the FOV comprises multiple vertical computational rays, which serve as the primary units of calculation for CUDA cores. These rays represent crucial data inputs from the 3D environment, essential for accurate sonar calculations. Data for each frame, including distances, normals, and object intersections, are collected for individual rays. Ray calculations are executed on separate CUDA cores to compute each beam, with beam patterns applied to weigh rays within the scene. Furthermore, beam interference within the FOV is analyzed to simulate speckle noise accurately. This approach enables efficient parallel processing and enhances the fidelity of the sonar simulation.

### 2.2. Previous Rasterized Scene Perception

**Figure 2:**
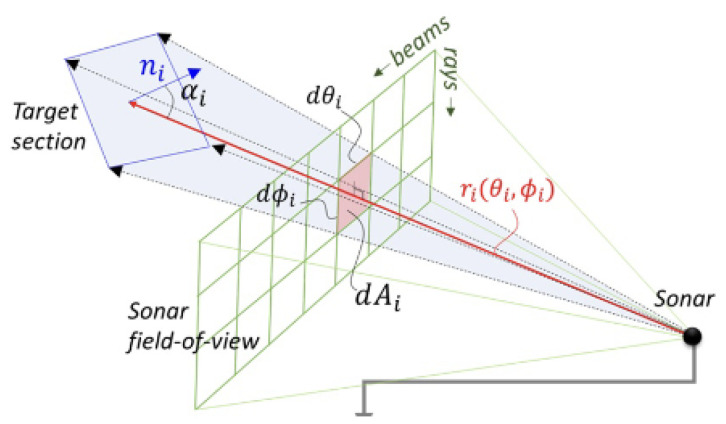
Schematic representation of a ray (the minimal calculation unit) that corresponds to a rasterized 2D plane grid pixel of the raster-based multibeam sonar [22].

In the previous methodology, a raster-based multibeam sonar plugin was developed to extend to Gazebo’s generic Depth Camera plugin. The original purpose of the Depth Camera plugin was to generate a 2D depth image of the scene, correlating each pixel with its corresponding distance from objects in the 3D world in alignment with the camera’s imagery. Consequently, the data acquired from the Depth Camera plugin are structured in a 2D rasterized grid format. This grid is characterized by uniform pixel dimensions in width and height within the 2D plane, as depicted in Figure 2.

**Figure 3:**
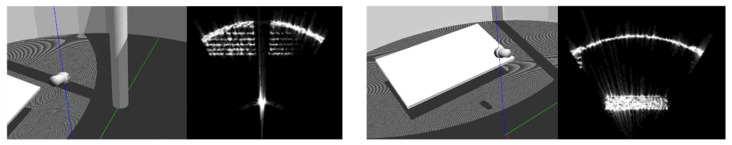
Sonar in a tank with plate and rod with sonar image generated by raster-based sonar simulations.

The raster-based, the Depth Camera-based, pipelining of the input data would result in low spatial resolution for the objects near the viewport’s edges. Also, it is discretized with equal vertical spacing in the rasterized plane, so the distance between rays would be larger at the edges of the grid. Moreover, the grid definition is set by the ratio of rows and columns. If the number of rows is set as a number of beams, the number of rays in each beam can not be modified. This is particularly problematic when the target object is large or the sonar is tilted down so that FOV is looking at the ground so that beams are grazing the ground. In such cases, more vertical rays are required to resolve the scene in the distance. In Figure 3, the sonar image is produced when the sonar is tilted downward in the tank. In the left figure, the ground imagery exhibits a deficiency in vertical ray data. This absence becomes less noticeable in the right figure, where the plate is closer to the sonar.

### 2.3. Ray-Based Scene Perception

**Figure 4:**
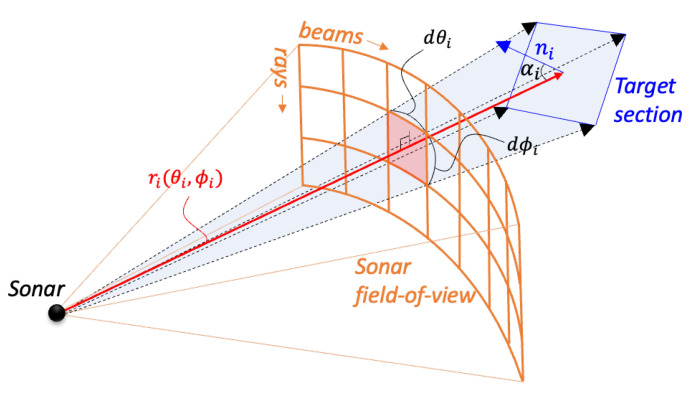
Schematic representation of a ray in the GPU Ray sensor that corresponds to an acoustic ray.

To address the limitations in vertical resolution encountered when the sonar is tilted downwards, the GPU Ray sensor is employed as an alternative to the Depth Camera sensor. This approach allows greater control over the number of vertical rays computed for each beam. Contrary to the Depth Camera sensor, which rasterizes and projects data onto a 2D plane, the GPU Ray sensor operates differently. In this setup, individual virtual rays are emitted from the sensor and intersect with objects in the virtual environment. The data from each virtual ray includes distance and normal information regarding the object it encounters. This method aligns closely with high-fidelity acoustic calculations, where each virtual ray is analogous to an acoustic ray, as depicted in Figure 4. Here, the ray-based multibeam sonar plugin, developed based on the Velodyne Simulator [27], facilitates the acquisition of point cloud datasets for sonar signal calculations.

**Figure 5:**
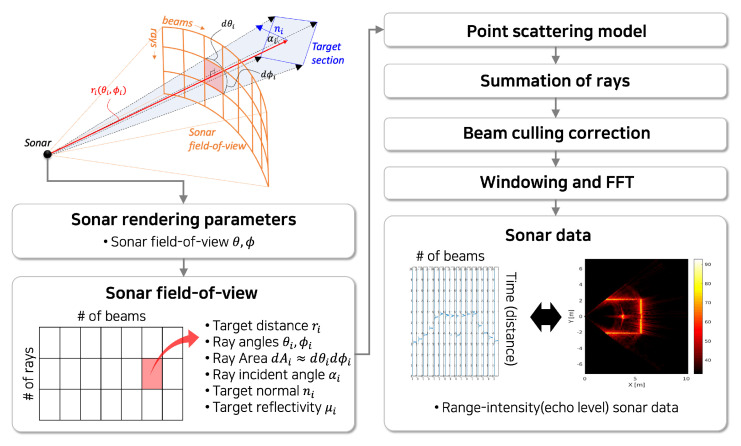
Overview of the Imaging Sonar Simulation Process. The process involves (i) capturing the underwater scene using a Gazebo GPU Ray plugin, (ii) gathering ray data from the rendered scene, (iii) developing an acoustic model for each ray, treating them as acoustic rays, (iv) combining rays into distinct beams, (v) assessing beam pattern effects on individual beams, and (vi) applying windowing and Fast Fourier Transform (FFT) techniques to produce range-intensity sonar data for each beam.

The fundamental procedure for sonar image simulation remains consistent, except for the initial data pipelining stage, which has been modified as Figure 5. Subsequent parameters are integrated for enhanced processing via CUDA parallel calculations.

### 2.4. Ray-Based Point Scattering Model

In the sonar image simulation, each ray is processed using the point-based scattering model as detailed in [24], where each ray matches each scatterer. These scatterers represent the intersection points of the rays with the object’s surface mesh.

The raw sonar acoustic signal spectrum for each beam, denoted as $P_{j}\left(f\right)$, is calculated using the following equation:

(1) $$P_{j}\left(f\right)=\left|S\left(f\right)\right|\sum_{n=1}^{N}\frac{a_{i}D\left(\theta_{i},\phi_{i}\right)e^{i\left(2\tilde{k}r_{i}\right)}}{{\left(r_{i}\right)}^{2}}$$

Here, the equation is formulated to incorporate the source spectrum |S(f)| that is emitted from the transducer of the sonar with beam directivity D(θ,ϕ) traveling in the direction of the $r_{i}$ with phase rotated as it travels, and the amplitude decreases with power of the distance it traveled and target’s reflected amplitude calculated as scatter amplitude $a_{i}$.

Distinct indices are utilized to characterize rays and beams. The index *i* is assigned to rays, with i=1,2,…N for a total of *N* rays, while $j=1,2,\ldots N_{B}$ denotes beams, where $N_{B}$ represents the total number of beams. The transmitted spectrum of the acoustic source is expressed as |S(f)|, and *N* signifies the total number of scatterers in the environment. Each scatterer is characterized by a complex amplitude $a_{n}$, and the acoustic frequency is denoted by *f*, measured in Hertz.

The complex wave number, $\tilde{k}$, comprises two parts: the real component $k_{w}$ and the imaginary component $k_{w}^{\prime }$. The real part is defined as $k_{w}=2\pi f/c$, where *c* represents the speed of sound in the medium, specifically seawater in this context. The imaginary part, $k_{w}^{\prime }$, is incorporated to account for attenuation effects in the propagation medium. This comprehensive representation of the wave number is crucial for accurately modeling the acoustic propagation in the underwater environment.

The equation for $P_{j}\left(f\right)$ combines the physical model for echo level and a complex random scale factor for speckle noise in the frequency domain. The acoustic frequency is given by *f* in Hertz. The beam index is denoted by subscript *j*. The beam directivity pattern, denoted as D(θ,ϕ), is a function of the azimuthal angle θ and elevation angle ϕ between the sensor and the scatterer. A simplified sinc function is commonly employed to model this beam directivity pattern. This directivity function represents the most intricate element of the model, as the actual directivity pattern of the hardware transducer that emits the beam is often not explicitly provided by manufacturers, and its design is frequently confidential. While the accurate representation of this pattern is critical for ensuring precise calculations and overall fidelity of the sonar simulation, the simplified sinc function is adopted here. This function is widely used in research and often provides excellent approximations, particularly for high-frequency transducers [28].

The source spectrum, a critical input parameter in the simulation model, is denoted as |S(f)| in the frequency domain and remains constant for all rays. A Gaussian model characterizes this spectrum by two essential parameters: the central frequency, $f_{c}$, and the bandwidth, *b*. These parameters determine the spectral position and width, respectively. This Gaussian representation offers a versatile and accurate model of the acoustic source, facilitating the simulation of diverse sonar systems with varying frequency characteristics. The source spectrum is mathematically expressed as follows:

(2) $$\left|S\left(f\right)\right|=S_{0}e^{-{\left(f-f_{c}\right)}^{2}b^{2}\pi^{2}}$$

While this Gaussian representation is not that of the standard Gaussian spectrum and is very simple, it is essential to incorporate more complex and realistic sonar transmission wave spectrums, along with appropriate sonar reception filters, in any practical simulation considering the specification of the sonar hardware. The source intensity, denoted by $|S_{0}|$, reflects the sonar’s source level, as detailed in [29].

The synthesis of time series data involves discretizing the acoustic frequency into a linear sequence from $f_{min}$ to $f_{max}$, centered around $f_{c}$. The bandwidth *b*, which represents the entire width of the transmitted spectrum, is a user-defined parameter aligned with the specifications of the simulated sonar system. For instance, the BlueView P900 Series Forward-Looking Sonar (FLS) utilizes a bandwidth of 2.95 kHz [30]. The frequency vector’s *m*-th element, where *m* is constrained to the interval [1, M], is defined by the following equation:

(3) $$f_{m}=m\Delta f+f_{min}$$

The frequency parameters are defined as $f_{min}=f_{c}-b/2$, with Δf=1/T representing the frequency spacing, and M=bT denoting the total number of frequencies. The variable *T* corresponds to the desired temporal duration of the signal. The wave number is computed for each frequency as $k_{m}=2\pi f_{m}/c$. Upon obtaining the frequency–domain response, the time–domain response is subsequently derived through an inverse Fourier transform, typically implemented using a fast Fourier transform algorithm. This methodology ensures a comprehensive representation of the acoustic signal in frequency and time domains, facilitating accurate simulation of sonar system responses. Also, it implemented generic FFT functions of the CUDA library.

For short-range acoustic propagation modeling, spherical spreading is considered a valid assumption. The model incorporates two-way transmission loss for incoherent scattering in $P_{j}\left(f\right)$. This loss is represented in the denominator by the term $2r_{i}^{2}$, where $2r_{i}$ signifies the round-trip distance. This formulation accurately accounts for the attenuation of acoustic energy as it propagates through the medium and reflects back to the sonar transducer, ensuring a more precise representation of the acoustic field in the simulation environment.

Each ray’s scattering amplitude, $a_{i}$, is computed and correlated with the target strength as delineated in Equation (1). The mathematical expression for $a_{i}$ is given by the following equation:

(4) $$a_{i}=\frac{\xi_{xi}+i\xi_{yi}}{\sqrt{2}}\sqrt{\mu_{i}\cos^{2}\left(\alpha_{i}\right)r_{i}^{2}d\theta_{i}d\phi_{i}}$$

In the formulation, $\xi_{xi}$ and $\xi_{yi}$ represent independent Gaussian random variables, with the subscript *i* denoting the ray index. These variables model Gaussian noise to fulfill the speckle noise requirement [24]. A coherent field component is incorporated into $a_{i}$ and complex Gaussian noise and coherent field results in Rician statistics [31]. The elements under the square root in the equation characterize the target strength of a ray upon impact with an object. This comprehensive approach ensures an accurate representation of various scattering phenomena and noise characteristics in the sonar simulation model.

## 3. Real-World and Simulated Sonar Data Comparisons

The deterministic sonar calculation using the point-scattering model in Equation (1) can be tuned to represent various sonar specs; the source level of the source spectrum |S(f)| and the directivity pattern D(θ,ϕ) of the each transducer is often not provided by the manufacturer. Therefore, to make best quantitative comparisons, the sonar with two operating frequency modes are used. First, the source level is calibrated using the one of the frequency mode by means of the simple directivity function (the sinc function). Thereafter, the operating frequency mode is changed with all other parameters fixed to validate intensity peak levels.

### 3.1. Experiment Descriptions

The experiments were conducted in a water tank measuring 92 inches in diameter with a water depth of 48 inches. The tank did not include acoustic linings for noise reduction. For sonar sensing, a Blueprint Oculus M1200d Multibeam Sonar (Blueprint Design Engineering Ltd., Ulverston, UK) was employed and later for 3D Scanning Sonar (NFEC-2025-02-303186), with detailed specifications provided in the Table 1. The target object used in the experiments was an Aluminum 6061 rod, measuring 4 inches in diameter and 7 inches in length. Two experimental configurations were used: a calibration case using high frequency (2.1 MHz) with the rod placed at 50 cm distance and 0 degree orientation, and a validation case using low frequency (1.2 MHz) with the rod at 50 cm distance and 15 degree orientation.

### 3.2. Calibration Case

**Figure 6:**
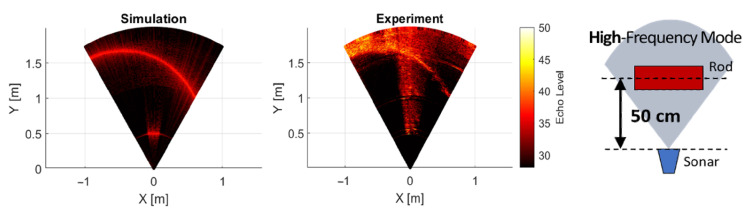
Simulated image (left) and experiment image (right) of the calibration case in high frequency operating mode.

**Figure 7:**
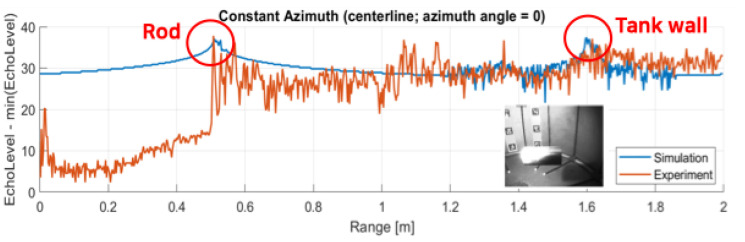
Comparison of echo intensity levels between simulation and experiment at the centerline for calibration case.

First the calibration of calculating parameter is conducted using the high frequency operating mode: 2.1 MHz mode with the rod placed at 50 cm distance and 0 degree orientation. The results of the image obtained is shown in Figure 6. Also, the echo intensity level at the centerline (0 azimuthal angle) is compared in Figure 7. Through these calibration case comparisons, the source level is set to 170 dB and the reflectivity of the Rod and Tank is set to 0.1 and 0.2, respectively, to match the peak levels of the rod and the tank. The experiment dataset is averaged using 100 image datasets out of 1100 images (about a second long recordings).

### 3.3. Validation Case

**Figure 8:**
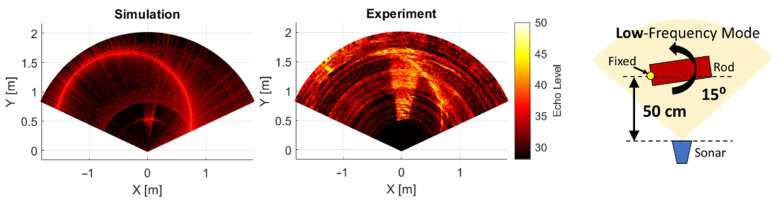
Simulated image (left) and experiment image (right) of the validation case in low frequency operating mode.

**Figure 9:**
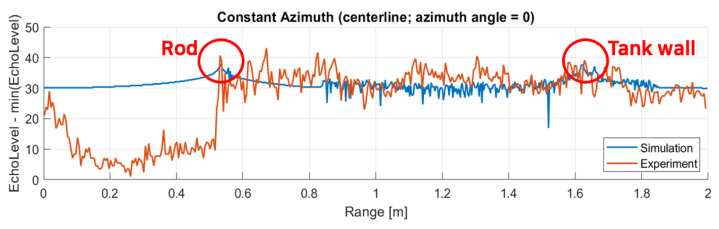
Comparison of echo intensity levels between simulation and experiment at the centerline for validation case.

Using the source level and the reflectivity parameters obtained from the calibration case, the validation is conducted. For the validation, the operation frequency mode is changed to 1.2 MHz to validate the frequency dependencies. Also, the orientation of the rod is tilted to 15 degrees to validate dependence of the incident angle to reflectivity and beam pattern. The results of the image obtained are shown in Figure 8. Also, the echo intensity level at the centerline (0 azimuthal angle) is compared in Figure 9.

The results show that the peak levels of the aluminum rod and the sonar tank wall well matched with the experiment. This validates that the underlying physical calculation using Equation (1) well represents the real-world acoustic characteristics even with the simplest directivity pattern when the object has sufficient reflectivity and compared to background environments in the scene.

## 4. Real-Time Simulation in ROS-Gazebo Framework

In this section, the results of ray-based and raster-based models are first compared for a relatively simple case (a front floating object) to confirm that the ray-based method maintains equal quality in sonar image generation. The version of the ROS and Gazebo used here are Noetic and version 11.5.1, respectively. This comparison demonstrates that implementing the new input data pipeline with equivalent physics calculations does not affect image quality. Second, the ground grazing local search scenario examination explicitly demonstrates the effectiveness of ray-based methods in sonar image generation. While the raster-based method shows clear limitations due to uncontrollable resolution constraints, the ray-based approach provides greater flexibility and can remedy such situations.

### 4.1. Comparisons for Front Floating Objects

**Figure 10:**
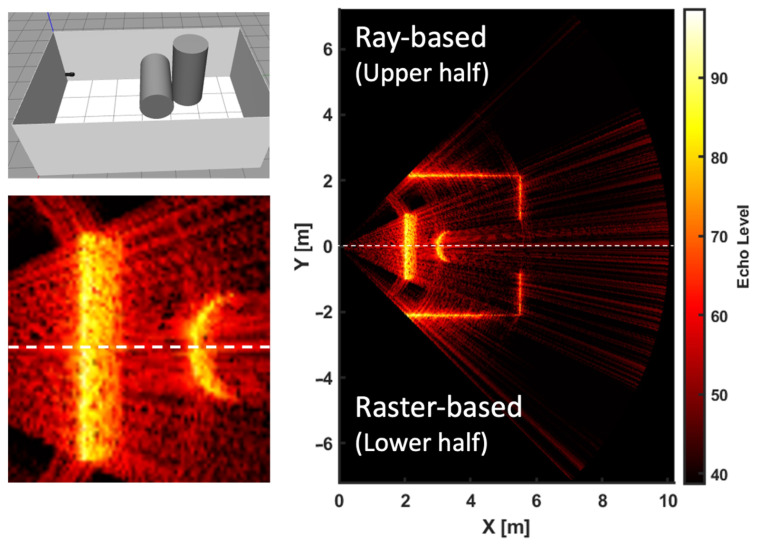
Comparative Analysis of sonar images using ray-based and raster-based models. The right section of the figure presents the overall comparison, while the bottom left provides a close-up view for detailed observation.

An initial comparative study was performed between raster-based and ray-based model plugins under identical virtual environment settings to confirm that the new method maintains the quality of the sonar image generation with physical characteristics correctly. This comparison involved positioning two cylinders in a square tank, one horizontally and the other vertically, in front of the sonar. The setup, depicted in Figure 10, was designed to evaluate the simulator’s imagery. The configurations used in the sonar simulation are detailed in Table 2. The results, as shown in Figure 10, effectively demonstrate the target object and the sonar tank, with both models providing clear visualization of beam scattering (the scattering effect, which is overlap interference between each beam’s beam patterns to adjacent beams, are shown as blazing tank walls). Here, the red-blazing sonar image is plotted using MATLAB (Version 2022a), manipulating the raw sonar image intensity data for each beam obtained from the model plugin as a ROS message topic.

The results depicted in Figure 10 showcase the target objects in the square tank. The images from the raster-based and ray-based models vividly highlight the beam scattering near the target cylinder. The refresh rate for the sonar images in both models was measured equally at 3 Hz, tested on a workstation equipped with an Intel i9-9900K 3.6 GHz processor and an Nvidia GeForce RTX 2080Ti graphics card. A significant observation is that the summation operation, being the most resource-intensive part of the process, dictates the maximum range capability. Optimization of ray count and reduction of maximum range facilitate the achievement of refresh rates surpassing 10 Hz on the specified hardware configuration. Furthermore, compared to the Depth Camera in Gazebo, the computational load added by the GPU Ray sensor appeared to be minimal, as indicated by the consistent refresh rates in both models. A key advantage of the Ray-based model is its flexibility in adjusting the number of rays while maintaining the equal quality of the sonar image to that of the raster-based model.

### 4.2. Comparisons for Local Search Scenario

The ray-based model’s adaptability in adjusting the number of rays presents a notable advantage, particularly in scenarios requiring high-resolution imaging at extended ranges, such as local search operations. This flexibility becomes especially valuable in specific underwater contexts, for example, when the sonar is oriented downward for bottom surface scanning with multiple objects on a sandy sea floor. In such scenarios, the ability to fine-tune the ray density allows for optimized resolution and detail capture, enhancing the detection and classification of objects on the seafloor. This feature enables a more accurate representation of complex underwater environments, facilitating improved performance in seabed mapping, object detection, and environmental monitoring tasks. The capacity to balance computational resources with imaging resolution through ray adjustment contributes significantly to the model’s efficacy across diverse underwater applications and varying operational requirements.

**Figure 11:**
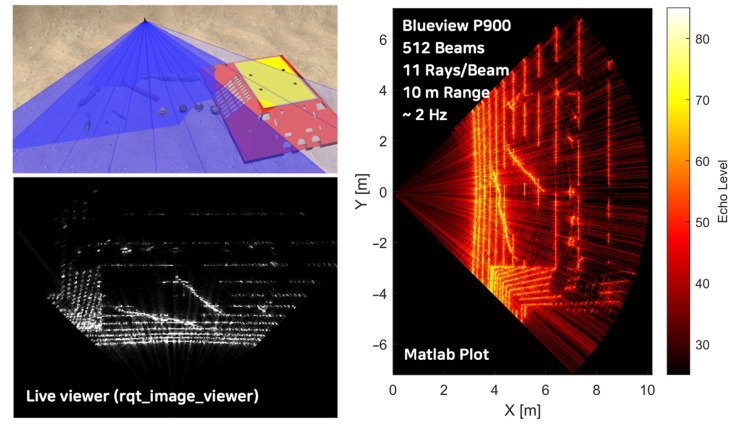
Local search scenario sonar image generated using the raster-based model.

**Figure 12:**
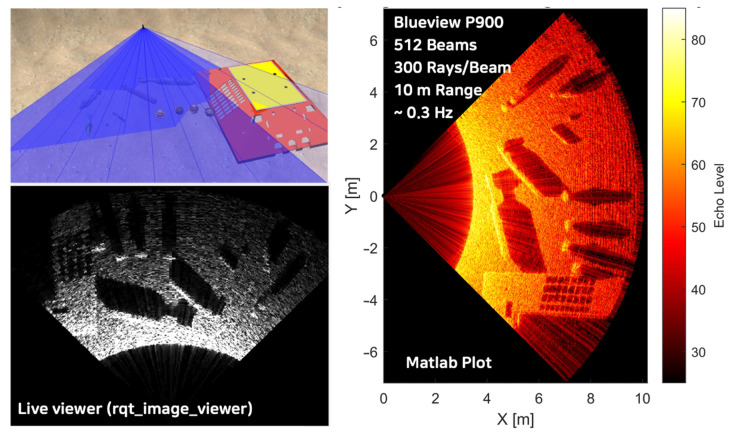
Local search scenario sonar image generated using the ray-based model.

Utilizing the BlueView P900 Series FLS with parameters specified in Table 2, the number of rays is adjusted to highlight the differences between models. The ray calculation iteration was constrained to 11 rays per beam in the raster-based model, resulting in a 2 Hz refresh rate (Figure 11). Conversely, the ray-based model was configured to incorporate 300 rays per beam (Figure 12), a configuration unattainable with the raster-based approach. This comparison underscores the enhanced capability and efficiency of the ray-based model in handling higher ray densities.

The raster-based model, with its limited number of rays, clearly exhibits gaps in the sonar image of the ground surface, making it unsuitable for local search scenario tests and evaluations. Conversely, by increasing the number of rays, the ray-based model provides sufficiently high resolution for robot control systems to recognize surrounding environments effectively. Although the refresh rate for the Blueview P900, set for a 10-meter range, drops to 0.3 Hz, it remains practically viable for simulating robotic sensors to test and evaluate the system integrity and performances (Table 3).

## 5. Conclusions

This research represents a significant advancement in the simulation of multibeam sonar for underwater robotics, highlighting the superiority of the ray-based approach over traditional raster-based methods. The comparative analysis conducted in this study demonstrates that the ray-based model matches the output quality of raster-based simulations and offers enhanced flexibility and adaptability, particularly in complex underwater scenarios. This flexibility proves crucial in high-resolution imaging tasks across varied ranges, such as local search scenarios.

A notable improvement facilitated by the ray-based method is evident in bottom-grazing viewpoint scenarios. In these challenging conditions, where the sonar beam interacts with the seafloor at shallow angles, the ray-based approach provides more accurate and detailed representations of the acoustic scattering. This enhancement is particularly valuable for applications such as seafloor mapping, obstacle avoidance in near-bottom operations, and detecting small objects partially buried in sediment. The ability to accurately simulate these complex acoustic interactions contributes significantly to the overall fidelity of underwater robotic simulations.

The integration of this model into the ROS-Gazebo framework, coupled with CUDA parallel computing, enables efficient real-time simulations. This computational efficiency reduces the reliance on costly and potentially hazardous sea trials, providing an invaluable tool for pre-experimental testing in underwater robotics. The ability to conduct extensive virtual trials under various environmental conditions and operational scenarios accelerates the development and refinement of autonomous underwater systems.

A key contribution of this research is the enhancement of sonar image simulation by adopting ray-based method instead of raster-based method. This transition represents a significant leap in simulation fidelity, allowing for a more accurate representation of complex acoustic phenomena. The ray-based approach enables the simulation of individual acoustic paths, accounting for factors such as refraction, reflection, and scattering with greater precision than previously possible with raster-based methods.

Furthermore, this study demonstrates that the ray-based method offers greater flexibility and applicability in diverse underwater scenarios than the raster-based approach. This enhanced versatility is particularly evident in complex geometries, varying environmental conditions, and dynamic scenes. The ray-based model’s ability to adapt to these diverse scenarios makes it a more robust and reliable tool for simulating a wide range of underwater robotic operations.

In conclusion, this study’s ray-based physical modeling approach constitutes a valuable contribution to underwater robotics. It significantly enhances the fidelity and usability of sonar-based simulations, paving the way for more sophisticated and reliable autonomous underwater systems. The improved accuracy in bottom-grazing scenarios, coupled with the overall flexibility of the ray-based approach, addresses critical challenges in underwater perception and navigation.

Future research directions will focus on further optimizing this model for various underwater scenarios, incorporating diverse directivity models and real-world hardware specifications. These enhancements will broaden the model’s applicability and efficiency in real-world applications. Additionally, efforts will be made to validate the produced sonar image quantitatively with experiment results. This ray-based sonar simulation model’s continued refinement and application promise to accelerate innovation in underwater robotics, contributing to advancements in fields such as ocean exploration, marine conservation, and underwater infrastructure inspection.

## Figures and Tables

**Table 1:** Blueprint Oculus M1200d hard specifications and parameters.

Parameter	Specification	Unit
Frequency (LF/HF)	1.2/2.1	MHz
Max Range (LF/HF)	30/10	m
Min Range	0.1	m
Update Rate (max)	40	Hz
Horizontal Aperture (LF/HF)	130/60	Degrees
Vertical Aperture (LF/HF)	20/12	Degrees
Number of Beams	512	-

**Table 2:** Blueview P900-90 hard specifications and parameters.

Parameter	Specification	Unit
Frequency	900	kHz
Bandwidth	2.95	kHz
Field-of-View	90	Degrees
Range	10	m
Beam width	1 × 20	Degrees
Beam spacing	0.18	Degrees
Number of beams	512	-
Number of rays	228	-
Source level	220	dB re μPa

**Table 3:** Comparisons of computational costs for local search scenario.

Methods	Range [m]	Number of Rays [-]	Ray Signal [s]	Summation [s]	Correction [s]	FFT [s]	Refresh Rate [Hz]
Raster	10	11	0.004	0.04	0.01	0.004	2
Ray	10	300	0.9	2.31	0.01	0.01	0.3

## Data Availability

The multibeam sonar model described in this paper is developed on top of the open-source Dave project using the ROS-Gazebo framework, available at https://github.com/Field-Robotics-Lab/dave and https://github.com/Field-Robotics-Lab/nps_uw_multibeam_sonar (accessed on 25 February 2025).

## Abbreviations

The following abbreviations are used in this manuscript:

ROS	Robot Operating System
GPU	Graphics Processing Unit
FFT	Faster Fourier Transform
LF	Low Frequency
HF	High Frequency
